# Supplementary material for: Single molecule analyses reveal dynamics of Salmonella translocated effector proteins in host cell endomembranes
Source: Nat Commun. 2023 Mar 4;14:1240. doi: 10.1038/s41467-023-36758-9 (PMC9985595; doi:10.1038/s41467-023-36758-9)
Supplement: Supplementary file 1 — Supplementary Information [file 41467_2023_36758_MOESM1_ESM.pdf]

Supplementary Information

Single molecule analyses reveal dynamics of *Salmonella* translocated effector proteins in host cell endomembranes

Vera Göser, Nathalie Sander, Marc Schulte [*], Felix Scharte [*], Rico Franzkoch [*], Viktoria Liss, Olympia E. Psathaki, and Michael Hensel

This PDF file includes:

Supplementary Materials and Methods

Supplementary Tables 1 to 2

Supplementary Figs. 1 to 12

Suppl. Materials and Methods

Western blot analysis

STM strains were cultured overnight (o/n) in synthetic minimal medium PCN [1] (phosphate, carbon nitrogen), 1 mM P_i, pH 7.4 medium, diluted 1:31 in fresh PCN, 0.4 mM P_i, pH 5.8 medium and subcultured for 6 h. Subsequently, the optical density at 600 nm (OD_{600}) was measured and 300 µl of bacterial suspension was transferred to a 1.5 ml tube. Bacteria were pelleted by centrifugation (22,000 x g, 2 min, 4 °C), resuspended in 1 x SDS sample buffer adjusted to 1 unit of OD_{600} per 100 µl and lysed by incubation at 100 °C for 5 min. Samples of 10 µl were subjected SDS-PAGE at 150 V for 75 min. Semi-dry blotting onto 0.45 µm nitrocellulose membranes (Amersham by Merck, GE10600002) was performed at 10 V constant for 45 min. Blocking of membranes was performed with 5% milk powder in TBS/T (0.1% Tween 20 in TBS) for 60 min at RT. Subsequently, primary antibody against HA tag was incubated (1:10,000 in TBS/T) for 1 h at RT, followed by incubation of secondary HRP-coupled antibody (1:10,000 in TBS/T) for 1 h at RT. Antibodies used in this study are listed in Supplementary Table 2. Between incubation with antibodies, membranes were washed thrice with TBS/T for at least 10 min each. Membranes were incubated ECL detection reagent (Amersham by VWR, RPN22329) and chemiluminescence signals were recording using the ChemiDoc system from BioRad and its corresponding software ImageLab.

DAB photo-conversion for EM

The illumination of the green fluorescent flavoprotein miniSOG (mini Singlet Oxygen Generator) was shown to generate sufficient singlet oxygen to locally catalyze the polymerization of diaminobenzidine (DAB, Sigma-Aldrich, D8001) by photo-conversion into an osmiophilic reaction product resolvable by EM [2]. We generated constructs for mammalian expression of LAMP1-miniSOG-mCherry (p3805) and LAMP1-tdminiSOG-mCherry (p3806) constructs, which after expression both located inside the lumen of endosomes/lysosomes. With these tools, vesicle fusion events could be visualized by CLEM.

Therefore, HeLa cells were grown on gridded MatTek dishes (MatTek Corporation, P35G-2-14-CGRD), transfected with LAMP1-miniSOG-mCherry or LAMP1-tdminiSOG-mCherry, infected with STM WT strain and at 8 h p.i. observed by live-cell imaging before fixation on stage with 2.5% glutaraldehyde (Science Services, E16216) in HEPES buffer (0.2 M HEPES Sigma-Aldrich, H-4034, pH 7.4, 5 mM $CaCl_2$, Sigma-Aldrich, C5670) for 1 h. After fixation cells were rinsed

several times in buffer containing 50 mM glycine (Biomol, 04943.1) and 20 mM potassium cyanide (Sigma-Aldrich, 60178), to reduce unspecific DAB staining, followed by rinses in buffer only. Photo-conversion of DAB was conducted as described before [3]. In short, fixed cells expressing LAMP1-miniSOG-mCherry were covered with freshly-prepared ice-cold 1 mg x ml^{-1} DAB in 0.2 M HEPES buffer. The ROI was relocated by CLSM and DAB photo-conversion was started by irritating the ROI with blue light (Xenon lamp, full power) until a brown DAB polymer was visible by eye. Afterwards, the DAB solution was removed and the cells were washed several times in HEPES buffer. Subsequently, the samples were further processed for TEM as described in main Methods section.

CLEM with high-pressure freezing and freeze substitution

HeLa cells stably transfected with LAMP1-GFP were grown on 3 mm sapphire disks (Engineering Office M. Wohlwend GmbH, Sennwald, Switzerland, 405) coated with 0.01% poly-L-lysine (Sigma-Aldrich, P8920) and a 15 nm thin gold imprint of a finder grid (Plano-EM, NHF15N) sputtered on top. Cells were infected with *Salmonella* Typhimurium and live-cell imaging (LCI) was performed at 6 h p.i. using an Olympus FV-1000 (Olympus, Hamburg, Germany). Directly after LCI cells were cryofixed by high-pressure freezing (HPF). For HPF sapphire disks were dipped into 1-hexadecene (Merck, 8.22064.0500) and placed with the cells facing upwards onto a flat aluminum planchette (diameter 3 mm) which was then covered with another aluminum planchette (diameter 3 mm, cavity 40 µm). The assembly was placed in the HPF holder and immediately frozen using a Wohlwend HPF Compact 03 (Engineering Office M. Wohlwend GmbH). Frozen samples were stored in liquid nitrogen until freeze substitution (FS). For FS aluminum planchettes (Engineering Office M. Wohlwend GmbH, 242) were opened in liquid nitrogen and separated from sapphire disks. Sapphire disks were transferred to FS solution containing 1% osmium tetroxide (Science Services, E19134), 0.1% uranyl acetate (Merck, 8473) and 5% H_2O in anhydrous acetone (VWR, 83683.230) pre-cooled to −90 °C. FS was performed in a Leica AFS2 (Leica, Wetzlar, Germany) following the protocol of 27 h at -90 °C, 12 h at -60 °C, 12 h at -30 °C and 1 h at 0 °C, washing with anhydrous acetone, embedding in EPON 812 (Sigma-Aldrich, 45359) mixed with acetone (30%, 60%, 100% EPON) and finally polymerization for 48 h at 60°C. Sapphire discs were separated from polymerized EPON block. Cells of interest were relocated on the EPON surface by means of the gold finder grid imprint. Ultrathin sections of 70 nm were cut with a Leica UC7 ultramicrotome, collected on formvar-coated grids (Plano-EM,

R1202) and post-stained for 30 min with 2% uranyl acetate and 20 min in 3% lead citrate (Science Services, E17800) and analyzed with a JEM 2100-Plus (JEOL, Japan) operating at 200 keV.

Cryo sample preparation for Tokuyasu immunogold labeling and TEM

Two days prior to infection HeLa LAMP1-GFP cells (1.5 x 10^6) were seeded into a 60.1 cm^2 Petri dish. cells were pre-fixed at 8 h p.i. for 10 min. with pre-warmed double-concentrated fixative (4% (w/v) PFA, Science Services, 15710, 0.2% (v/v) GA) in 0.1 M PHEM buffer 120 mM PIPES Sigma-Aldrich, P-6757, 4 mM $MgCl_2$ Merck, 1.05833.1000, 50 mM HEPES, 20 mM EGTA AppliChem, A0878) which was added to the culture dish 1:1 mixed with the culture medium. Subsequently, fixative was replaced by fresh fixative (2% (w/v) PFA, 0.1% (v/v) GA) and cells were fixed for 2 h at RT and stored o/n in 1% formaldehyde (w/v). Next, cells were washed twice with 0.1% glycine in 0.1 M PHEM, several times with 0.1 M PHEM buffer, scraped in PHEM containing 1% (w/v) gelatin (Sigma-Aldrich, G9391) from the culture plates and were pelleted by centrifugation (300 x g, 3 min). The cell pellet was infiltrated at 37 °C stepwise in 2% (w/v), 5% (v/w) and finally in 10% (w/v) gelatin in 0.1 M PHEM buffer. After gelation at 4 °C, 1 mm^3 cubes were dissected and infiltrated in 2.3 M sucrose (Biomol, 21986) o/n at 4 °C in rotating vials. Gelatin cubes were mounted on specific aluminum specimen holders and plunge-frozen in liquid nitrogen. Specimen holders were placed into the cryo-chamber of a cryo-ultramicrotome UC7 (Leica Microsystems, Wetzlar), precooled to -110 °C and trimmed to suitable block size. Ultrathin sections of 60 nm were cut at -110 °C with a dry cryo-immuno diamond knife (Diatome, Switzerland). Ribbons of sections were picked up with a wire loop filled with a 1+1 mixture of 1% (w/v) methyl cellulose (Sigma-Aldrich, M6385) and 2.3 M sucrose in PHEM buffer. Sections were thawed on the pick-up solution and transferred downwards to Formvar carbon-coated 100-mesh copper grids (Plano-EM, 09D00903).

For immunolabeling sections were placed 30 min on 37 °C warm water to diffuse pick-up solution and gelatin. Subsequently, grids were rinsed over a series of droplets: washed in 0.1% glycine in PBS, blocked 3 min in 1% BSA (Sigma-Aldrich, A0281) in PBS, incubated 60 min. in primary antibody diluted in 1% BSA, 0.2% fish skin gelatin (Sigma-Aldrich, G7765) in PBS, washed in 0.1% BSA in PBS, incubated 30 min in bridging antibody, diluted in 1% BSA, 0.2% fish skin gelatin in PBS, washed in 0.1% BSA in PBS , incubated 20 min in 10 nm protein A-gold diluted in 1% BSA in PBS, washed in PBS, fixed 5 min in 1% (v/v) glutaraldehyde in PBS and washed in distilled water. Sections were stained 5 min on drops of 2% uranyl oxalate (pH 7.0), shortly

rinsed in distilled water and incubated 10 min on drops of a mixture of 1.8% (v/w) methyl cellulose/0.4% uranyl acetate (pH 4.0) on ice. Finally, grids were looped out, most of the viscous staining solution drained away and sections dried in the residual thin film which covers the grid. The sections were analyzed using systems JEM 2100 Plus at 200 keV and Zeiss TEM 902 at 80 keV. Labeling were controlled and imaged at same regions on three following sections on each grid.

References:

1 Neidhardt, F. C., Bloch, P. L. & Smith, D. F. Culture medium for enterobacteria. *J Bacteriol* **119**, 736-747 (1974).
2 Shu, X. *et al.* A genetically encoded tag for correlated light and electron microscopy of intact cells, tissues, and organisms. *PLoS Biol* **9**, e1001041 (2011). https://doi.org:10.1371/journal.pbio.1001041
3 Krieger, V. *et al.* Reorganization of the endosomal system in *Salmonella*-infected cells: the ultrastructure of *Salmonella*-induced tubular compartments. *PLoS Pathog* **10**, e1004374 (2014). https://doi.org:10.1371/journal.ppat.1004374
4 Obert, S., O'Connor, R. J., Schmid, S. & Hearing, P. The adenovirus E4-6/7 protein transactivates the E2 promoter by inducing dimerization of a heteromeric E2F complex. *Mol Cell Biol* **14**, 1333-1346 (1994).

Supplementary Tables

Supplementary Table 1: Oligonucleotides and synthetic DNA used in this study.

Designation	Sequence (5´ - 3´)
Vf-HA	TACCCATACGACGTCCCAGA
Vr-pWSK29-2	GGTACCCAATTCGCCCTATAGTGAGTCGTATTAC
1f pWSK29-PsteC	TATAGGGCGAATTGGGTACCGGATAGCAAGTACG
ATAGCG	
1r-HaloTag-HA	TGGGACGTCGTATGGGTAACCGGAAATCTCCAGA GTAGACAGC
Vf-SseF-3HA	CGCGCTTGGCGTAATCATGG
Vr-SseF3HA	CTTGCCGCTGACGGAATATG
SseF253-For	AGTTCTGATCATACATCTGGGGA
Seq-Rev	AGCGGATAACAATTTCACACAGGA
pipB2 SDM-CC For	atagagactgatGCTGAGACATTGAAAAGTAC
pipB2 SDM-CC Rev	gtcttcctgatcTTGTGTTTCATTTCTCCG
sseJ SDM CC For	aaaactaatgatGAAGAATTAAAAGAAAAATACCCC
sseJ SDM CC Rev	gtccagggcatcGTGAGCGATGCTTTCATC
sopD2 SDM CC For	caaaataatgacAGAGAAATTGATGCTATTCTAAG
sopD2 SDM CC Rev	agactcatcatcATCCTTATTTAATACATCAGAAAAC
sifA Del-331 Rev	GCCGCTTTGTTGTTCTGAGCG
Del-L16 For	GGCTCTGCGGCGTCTGCG

Synthetic DNA: sseF-3xHA

ATAACAGAACGAAATATGAAAATTCATATTCCGTCAGCGGCAAGTAATATAGTCGAT
GGTAATAGTCCTCCTTCCGATATACAAGCGAAGGAGGTATCGTTTCCTCCCCCTGAA
ATTCCAGCGCCTGGCACCCCCGCAGCCCCTGTGCTGCTTACGCCTGAACAAATAAGG
CAGCAGAGGGATTATGCGATACATTTTATGCAATACACTATTCGTGCGCTGGGTGCG
ACAGTCGTGTTTGGGTTATCGGTTGCTGCAGCGGTAATTTCTGGCGGGGCAGGATTA
CCCATTGCTATTCTTGCGGGGGCGGCGCTCGTGATTGCTATTGGGGATGCTTGCTGTG
CGTATCATAATTATCAATCGATATGTCAGCAAAAGGAGCCATTACAAACCGCCAGTG
ATAGCGTTGCTCTTGTGGTCAGTGCGCTGGCCTTAAAATGTGGGGCAAGTCTTAACT

GCGCTAACACCCTTGCTAATTGTCTTTCTTTATTAATACGTTCAGGAATCGCTATTTCT
ATGTTGGTTTTACCCCTACAGTTTCCACTGCCCGCGGCTGAAAATATTGCGGCCTCTT
TGGACATGGGGAGTGTAATTACCTCCGTTAGCCTGACGGCGATAGGTGCGGTACTGG
ATTATTGCCTTGCCCGCCCCTCTGGCGACGATCAGGAAAATTCTGTTGATGAACTTCA
TGCCGATCCCAGTGTGTTATTGGCGGAACAAATGGCAGCGCTCTGTCAATCTGCTAC
TACACCTGCATTAATGGACAGTTCTGATCATACATCTCGGGGAGAACCATACCCATA
CGACGTCCCAGACTACGCTGGCTATCCCTATGACGTCCCGGACTATGCAGGATCCTA
TCCATATGACGTTCCAGATTACGCTTAATCTAGAGCGGCCGCCACCGCGGTGGAGCT
CCAGCTTTTGTTCCCTTTAGTGAGGGTTAATTGCGCGCTTGGCGTAATCATGGTCATA
GCTGTTTCCTGTGTGAAAT

Supplementary Table 2: Antibodies and conjugates used in this study.

Antibody	Dilution	Source
Primary antibodies:		
Mouse anti M45	1:50	[4], custom-made
Rat anti HA c3 F10 IgG	1:10,000 for WB<br>1:500 for LM	Roche by Merck #1 1867431001
Mouse anti HA c16B12 IgG	1:50 for EM	Roche By Merck # 93019429
Rabbit anti *Salmonella* O, group B factors 1, 4, 5, 12	1:500 for LM	BD Difco # 228151
Secondary antibodies:		
Alexa Fluor 568 goat anti mouse	1:1,000 for LM	Invitrogen by ThermoFisher # A-11004
Goat anti rat IgG HRP	1:10,000 for WB	Jackson ImmunoResearch # 112-035-003
Goat anti rabbit IgG Cy5	1:1,000 for LM	Jackson ImmunoResearch # 111-175-144
Alexa Fluor 568 goat anti rat IgG (H+L)	1:1,000 for LM	Life Technologies by ThermoFisher # A-11077
Conjugates:		
Protein A gold 10 nm	1:50 for EM	Cell Microscopy Core, University Medical Center, Utrecht, NL

Supplementary Figures

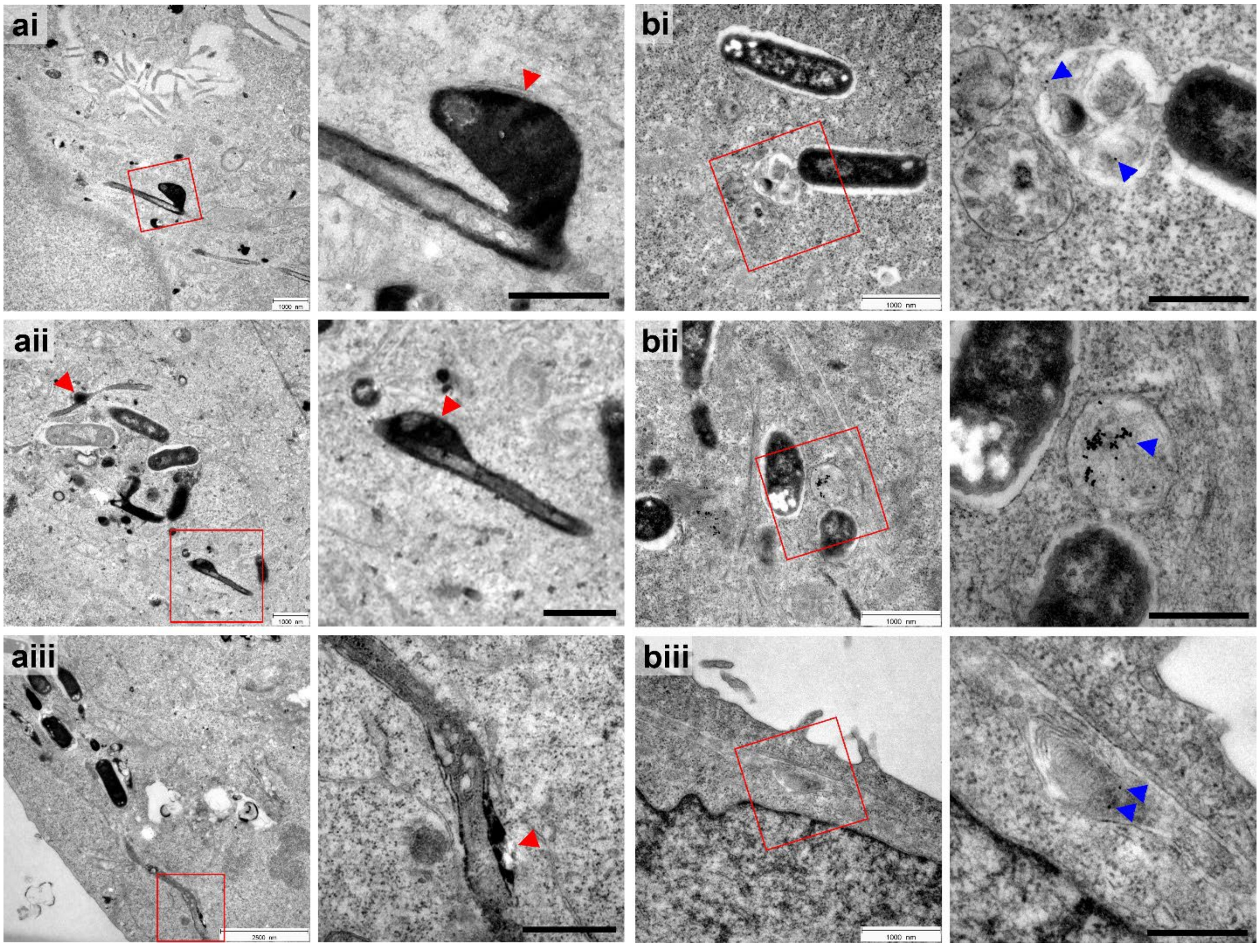

Supplementary Fig. 1. Delivery of vesicular content to SCV and SIF. a) Vesicular fusion of DAB-labeled endosomes/lysosomes with the SCV/SIF continuum. HeLa cells transiently expressing LAMP1-miniSOG-mCherry were infected with STM WT constitutively expressing GFP. At 6 h p.i. cells were microscopically checked for normal infection and SIF formation and subsequently fixed with 2.5 % GA. Photo-conversion of DAB was performed by means of miniSOG and cells were prepared for conventional EM as described in Methods. Notably, both the constructs LAMP1-miniSOG-mCherry and LAMP1-tdminiSOG-mCherry after expression ends up inside the lumen of endosomes/lysosomes. After chemical fixation, miniSOG is still functional and upon illumination photo-converts DAB to an osmiophilic polymer inside vesicles, which appear as electron-dense structure on EM sections. Panels **a**) show examples of different experiments. Arrowheads indicate fusion events of DAB-containing endosomes/lysosomes with dm SIF. **b**) Vesicular fusion of nanogold-labeled endosomes with the SCV/SIF continuum. HeLa

cells stably expressing LAMP1-GFP were infected with STM WT strain constitutively expressing mCherry. Pulse/chase with 10 nm colloidal gold conjugated with BSA-rhodamine was performed from 2-5 h p.i. At 7 h p.i. cells were microscopically checked for normal infection and SIF formation and subsequently fixed with 2.5 % GA and prepared for conventional EM as described in Methods. Blue arrowheads indicate gold particles. Micrographs show cells representative for three independent experiments. Scale bars: 1 µm (**a**i, **a**ii, **b**); 2.5 µm (**a**iii); 500 nm (**a**, **b** details).

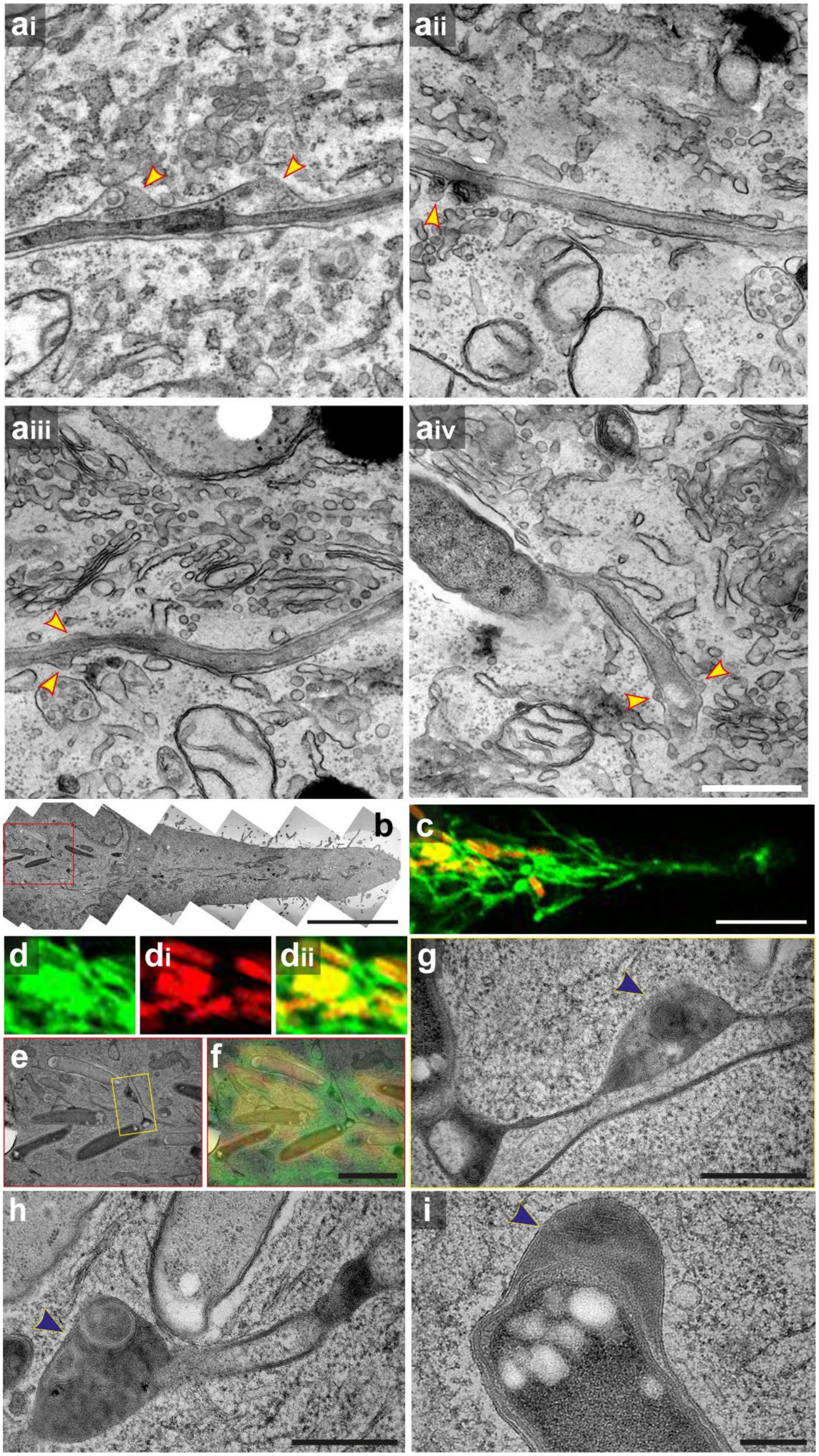
ai
aii
aiii
aiv
b
c
d
di
dii
e
f
g
h
i

Supplementary Fig. 2. Interactions of host cell endosomal membranes in STM-infected cells. HeLa cells stably expressing LAMP1-GFP were infected with STM WT strain constitutively expressing mCherry. At 8 h p.i. cells were checked by LM for normal infection and SIF formation. **a)** Cells were fixed with 2.5% GA and prepared for conventional EM as described in Methods. EPON sections of 70 nm containing infected cells with a SIF network were imaged by TEM. Panels in **ai** to **aiv** show representative examples for interaction of endosomal vesicles with SIF and SCV observed in independent experiments. Arrowheads indicate fusion events of vesicular structures with double membrane SIF. **b-i**) CLSM was performed to identify SIF-positive cells showing dynamic extension of SIF networks. After LCI, cells were prepared by HPF/FS for TEM as described in Methods. Correlation of CLSM and TEM modalities allowed identification of STM in SCV and extending SIF tubules (**d-g**). Arrowhead indicates fusion event of vesicular structure with dm SIF (**g**). Further fusion events were found at other positions (**h**, **i**). Micrographs show cells representative for three independent experiments. Scale bars: 10 µm (**b**, **c**); 2 µm (**d-g**); 500 nm (**a**, **g-i**).

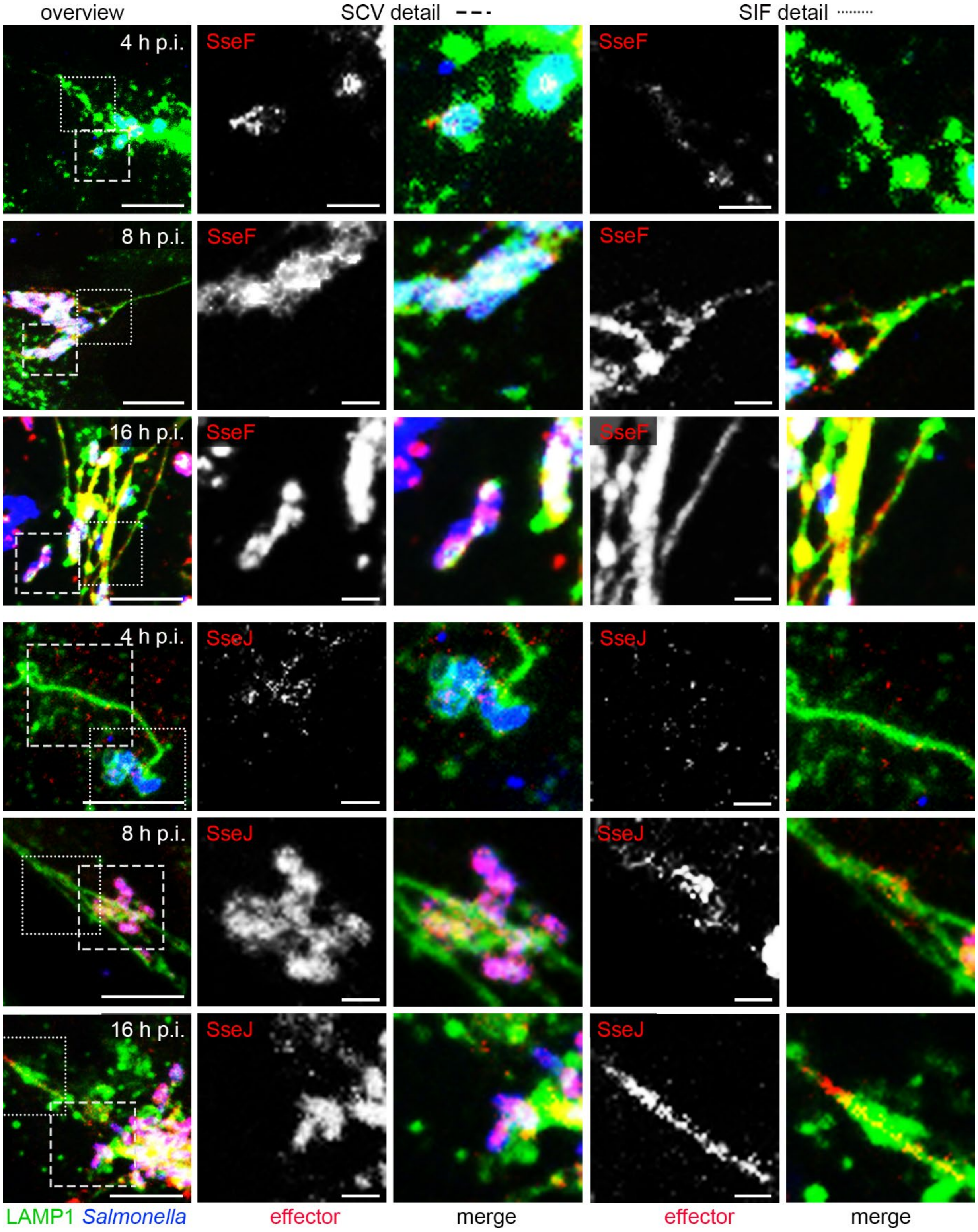

Supplementary Fig. 3. Distribution of translocated *Salmonella* SPI2-T3SS effector proteins over the course of infection. HeLa cells stably expressing LAMP1-GFP (HeLa LAMP1-GFP) were infected with STM WT expressing *sseF*::M45 or *sseJ*::M45 as indicated. At various time

points after infection, cells were fixed and immunolabeled for STM (blue) and effector proteins (red). Details of SCV (dashes boxes) and SIF (dotted boxes) are shown. Micrographs show cells representative for three independent experiments. Scale bars: 10 and 2 µm in overview and details, respectively.

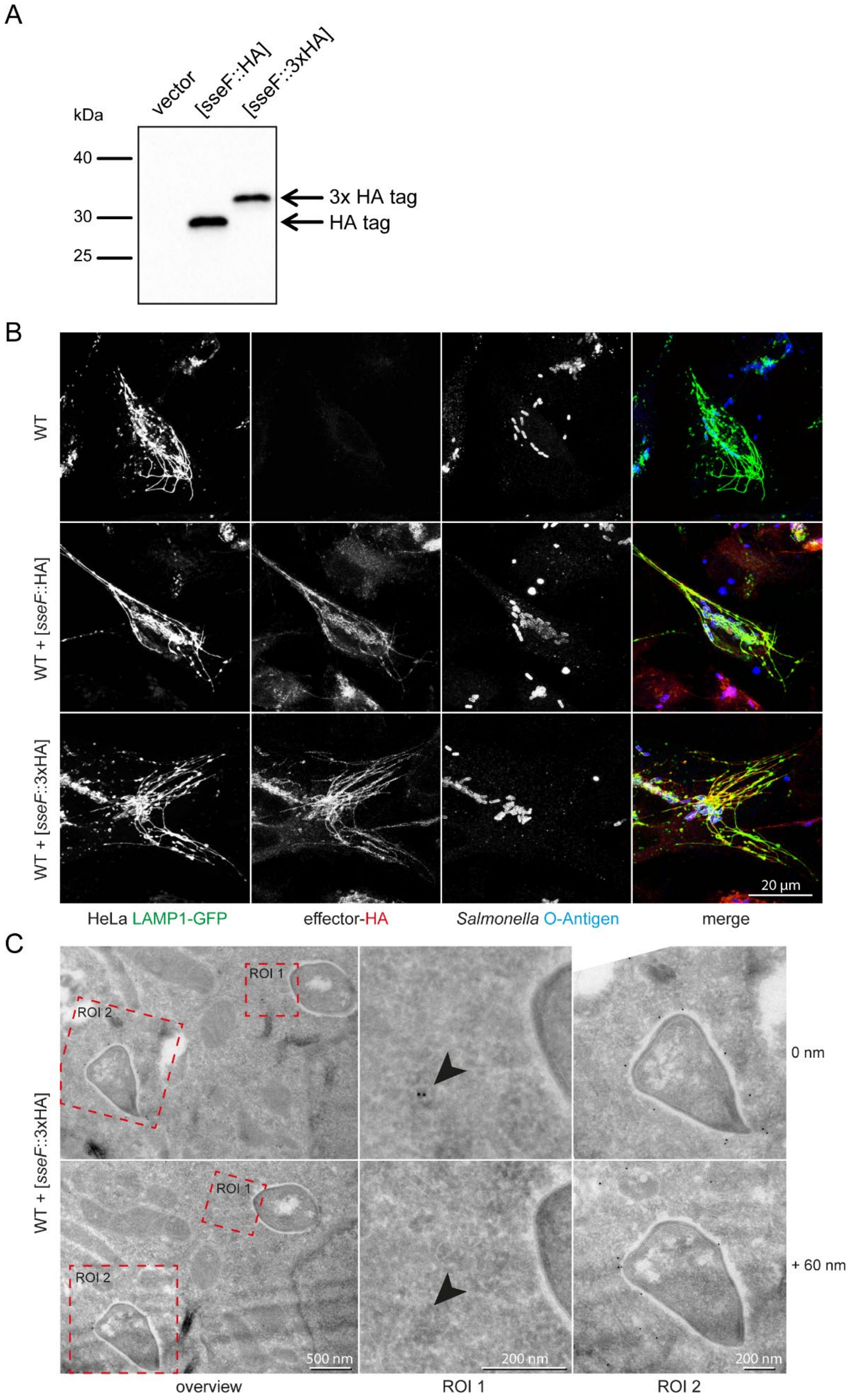
A
kDa
vector
[sseF::HA]
[sseF::3xHA]
40
30
25
3x HA tag
HA tag
B
WT
WT + [sseF::HA]
WT + [sseF::3xHA]
20 µm
HeLa LAMP1-GFP
effector-HA
Salmonella O-Antigen
merge
C
ROI 1
ROI 2
0 nm
+ 60 nm
WT + [sseF::3xHA]
500 nm
200 nm
200 nm
overview
ROI 1
ROI 2

Supplementary Fig. 4. Analysis of synthesis and translocation of triple-HA-tagged effector protein SseF. a) Protein synthesis of triple-HA-tagged SseF after 6 h growth of subcultures in PCN (0.4) pH 5.8 medium. All plasmids were investigated in *Salmonella* wild-type background. Separation was performed by SDS-PAGE, protein was transferred onto nitrocellulose membranes and epitope-tagged proteins were detected using a primary antibody against HA tag and a secondary HRP-coupled antibody. **b**) Translocation of triple-HA-tagged SseF into stably transfected HeLa LAMP1-GFP cells. Host cells were infected, fixed 8 h p.i. and immune-stained against HA epitope tag and O-antigen. Micrographs show cells representative for three independent experiments. Scale bar: 20 µm. **c**) Consecutive ultrathin sections of immunogold-labeled infected HeLa cells with STM WT expressing triple-HA-tagged SseF. Scale bars: 500 nm, 200 nm and 200 nm for overview, ROI 1 and ROI 2, respectively. Details (inserts ROI 1 and 2 in overviews) of HA-tagged SseF immunogold labeling are shown on two consecutive 60 nm thick sections. On first section (0 nm, ROI 1) immunogold is located inside a vesicle (s. arrowhead), clearly indicating for a vesicular structure also proven by the following section (+60 nm, ROI 1) missing the immunogold labeling at identical region (s. arrowhead) which proves for a non-tubular structure. HA-tagged SseF immunogold labeling shown in ROI 2 on first (0 nm) and consecutive section (+60 nm) support our specific immunogold labeling for SseF.

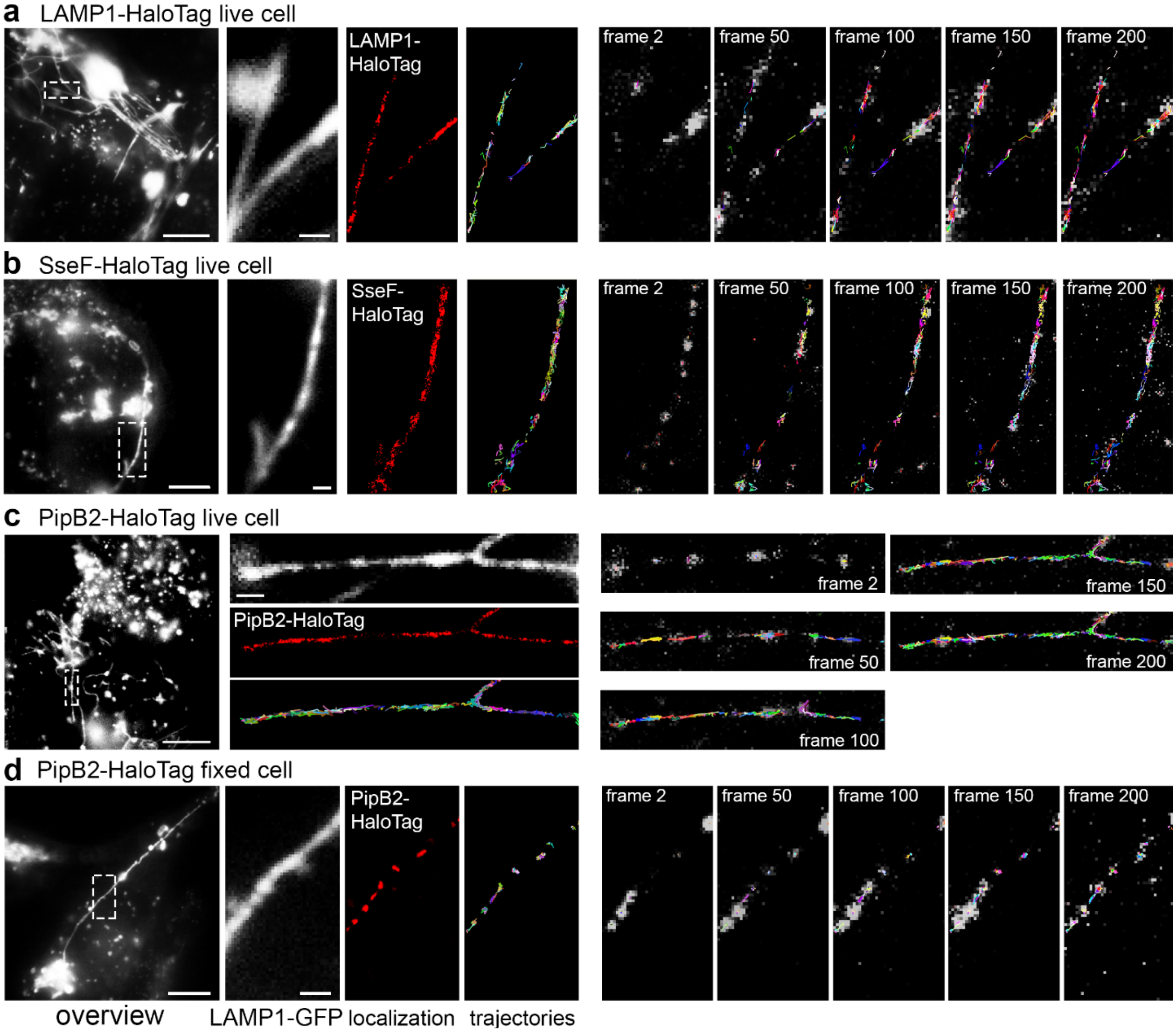

Supplementary Fig. 5. Single molecule localization (SML) and single molecule tracking (SMT) of effector proteins on double-membrane SIF. HeLa LAMP1-GFP cells were infected with *Salmonella* WT or mutant strains expressing various SPI2-T3SS effector proteins fused to the HaloTag at a multiplicity of infection (MOI) of 75. For visualization of LAMP1-HaloTag, cells were transfected for expression of LAMP1::HaloTag::HA one day before infection. Following incubation for 7 h under standard cell culture conditions, live cell imaging (LCI) was performed. Labeling reactions were performed directly bevor imaging using HTL-TMR with a final concentration of 20 nM for 15 min at 37 °C. SML images representative for three independent experiments are shown for LAMP1-HaloTag (**a**), SseF-HaloTag (**b**), PipB2-HaloTag (**c**) in living cells, and PipB2-HaloTag in fixed cells as control (**d**). Microscopy was performed using 15% laser power at the focal plane, at 32 frames per second, SML and SMT was rendered within 200

consecutive frames. Selected frames of the TMR signal, localization and tracking are presented, also showing elapsed trajectories. Sequences of 200 frames of effector proteins or LAMP1 fused to HaloTag are shown in Supplementary Movies 3, 4, 5, 6 corresponding to panels **a**, **b**, **c**, and **d**, respectively. Scale bars: 10 and 1 μm in overviews and details, respectively.

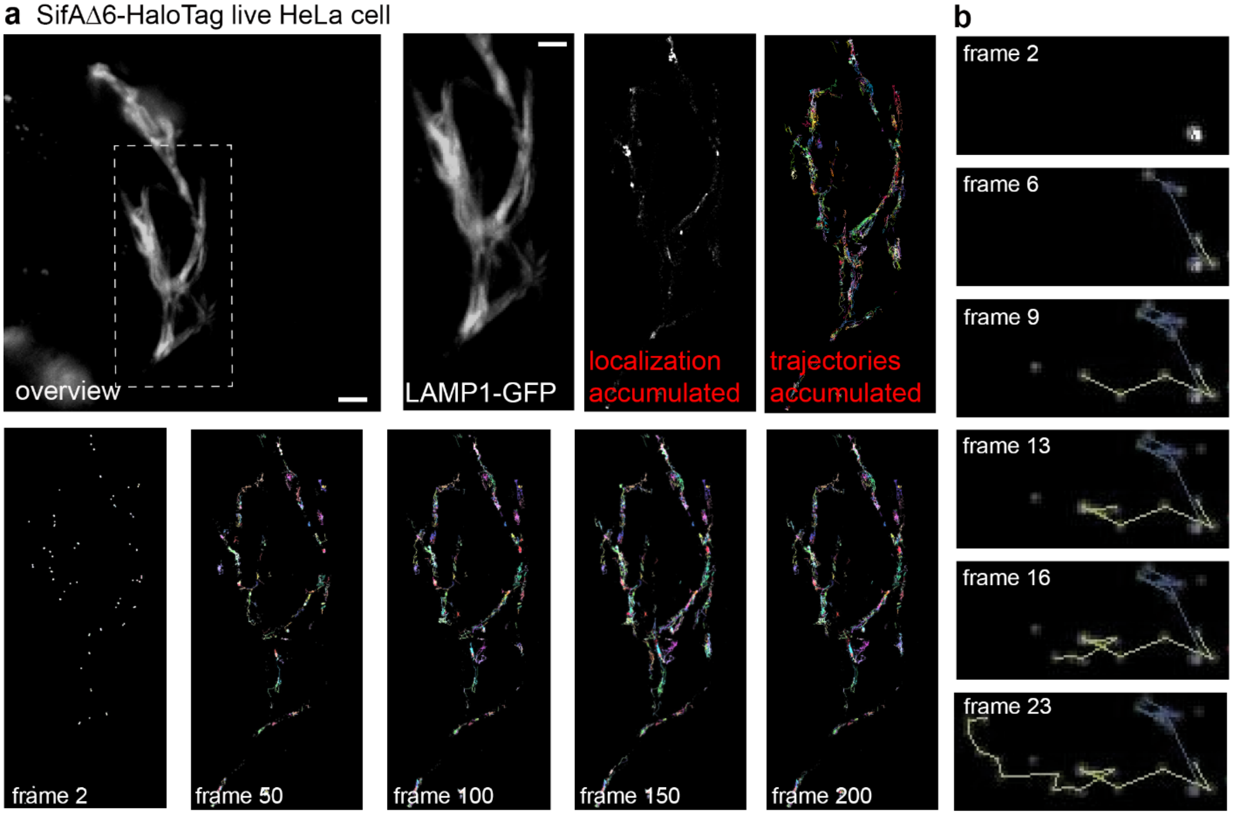

Supplementary Fig. 6. SML and SMT analyses of mutant alleles of effector proteins. Corresponding to **Fig. 4c.** HeLa cells stably expressing LAMP1-meGFP were infected with STM *sifA* mutant strain expressing *sifA*Δ6::HaloTag::HA at MOI 50. At 7 h p.i. LCI was performed. Labeling reactions were performed directly bevor imaging using HaloTag-TMR with a final concentration of 20 nM for 15 min at 37 °C. **a**) Shown are SRM images representative for three independent experiments acquired using 15 % laser power at the focal plane, rendered from SML and trackings within 750 consecutive frames were analyzed. **b**) Selected frames (frame rate: 32 frames per second) of *sifA*Δ6::HaloTag::HA TMR signal, localizations and trackings (also showing elapsed trajectories) are presented. Each trajectory has a different color. Scale bar, 10 µm.

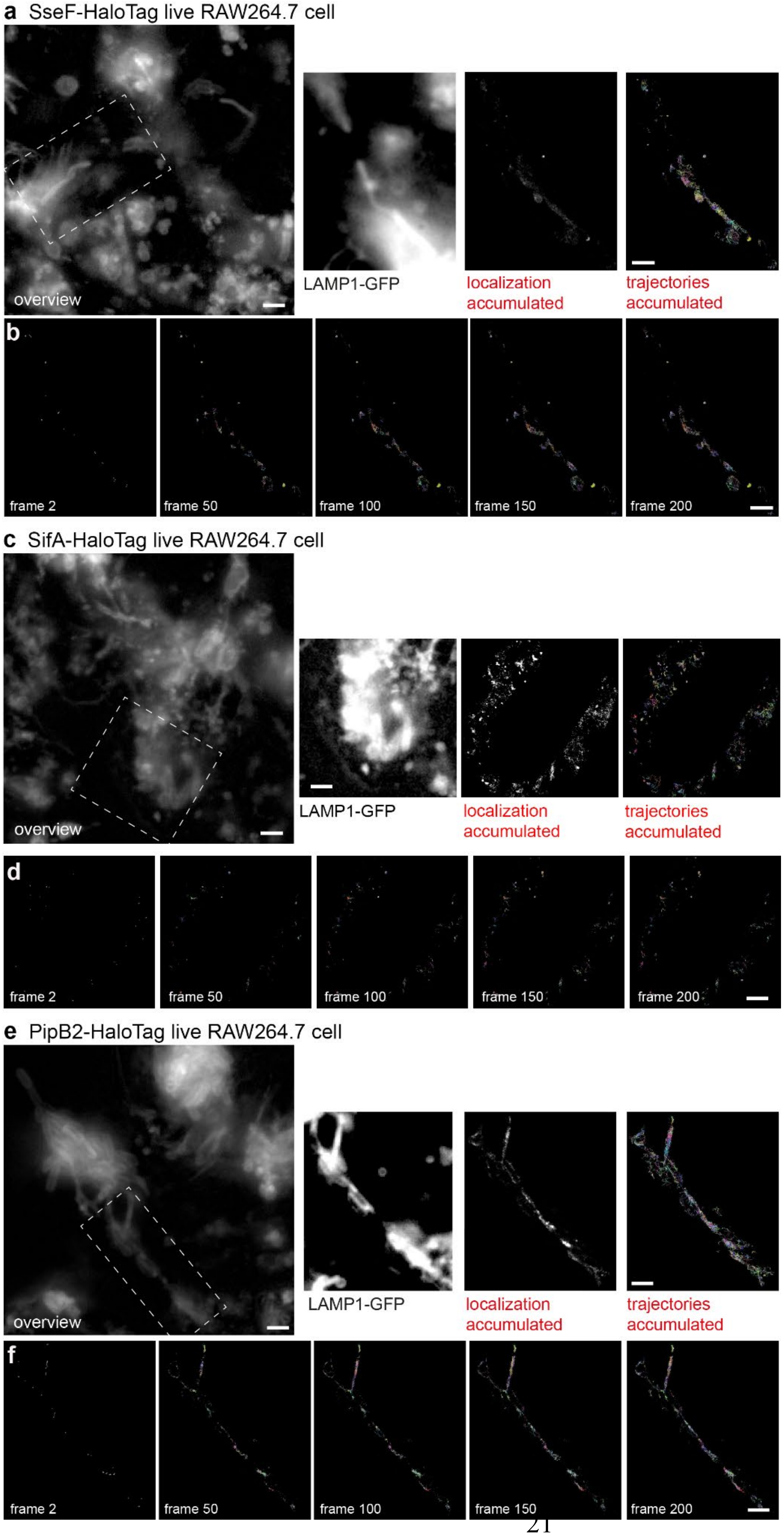
a SseF-HaloTag live RAW264.7 cell
overview
LAMP1-GFP
localization accumulated
trajectories accumulated
b
frame 2
frame 50
frame 100
frame 150
frame 200
c SifA-HaloTag live RAW264.7 cell
overview
LAMP1-GFP
localization accumulated
trajectories accumulated
d
frame 2
frame 50
frame 100
frame 150
frame 200
e PipB2-HaloTag live RAW264.7 cell
overview
LAMP1-GFP
localization accumulated
trajectories accumulated
f
frame 2
frame 50
frame 100
frame 150
frame 200

Supplementary Fig. 7. SML and SMT analyses of effector proteins in murine macrophage cell line RAW264.7. Corresponding to **Fig. 4d.** RAW264.7 cells stably expressing LAMP1-GFP were infected with STM strains at MOI 50. **a, b)** STM Δ*sseF2* expressing *sseF*::HaloTag::HA. **c, d**) STM Δ*sifA* expressing *sifA*::HaloTag::HA. **e, f**) STM Δ*pipB2* expressing *pipB2*::HaloTag::HA. Labeling reactions were performed directly bevor imaging using HaloTag-TMR with a final concentration of 20 nM for 15 min at 37 °C, and LCI was performed at 12 h p.i. Shown are SRM images representative for three independent experiments, acquired using 15 % laser power at the focal plane, rendered from single molecule localizations and trackings within 750 consecutive frames were analyzed (**a, c, e**). Selected frames (frame rate: 32 frames per second) of effector::HaloTag::HA TMR signal, localizations and trackings (also showing elapsed trajectories) are presented (**b, d, f**). Each trajectory has a different color. Scale bar 10 µm.

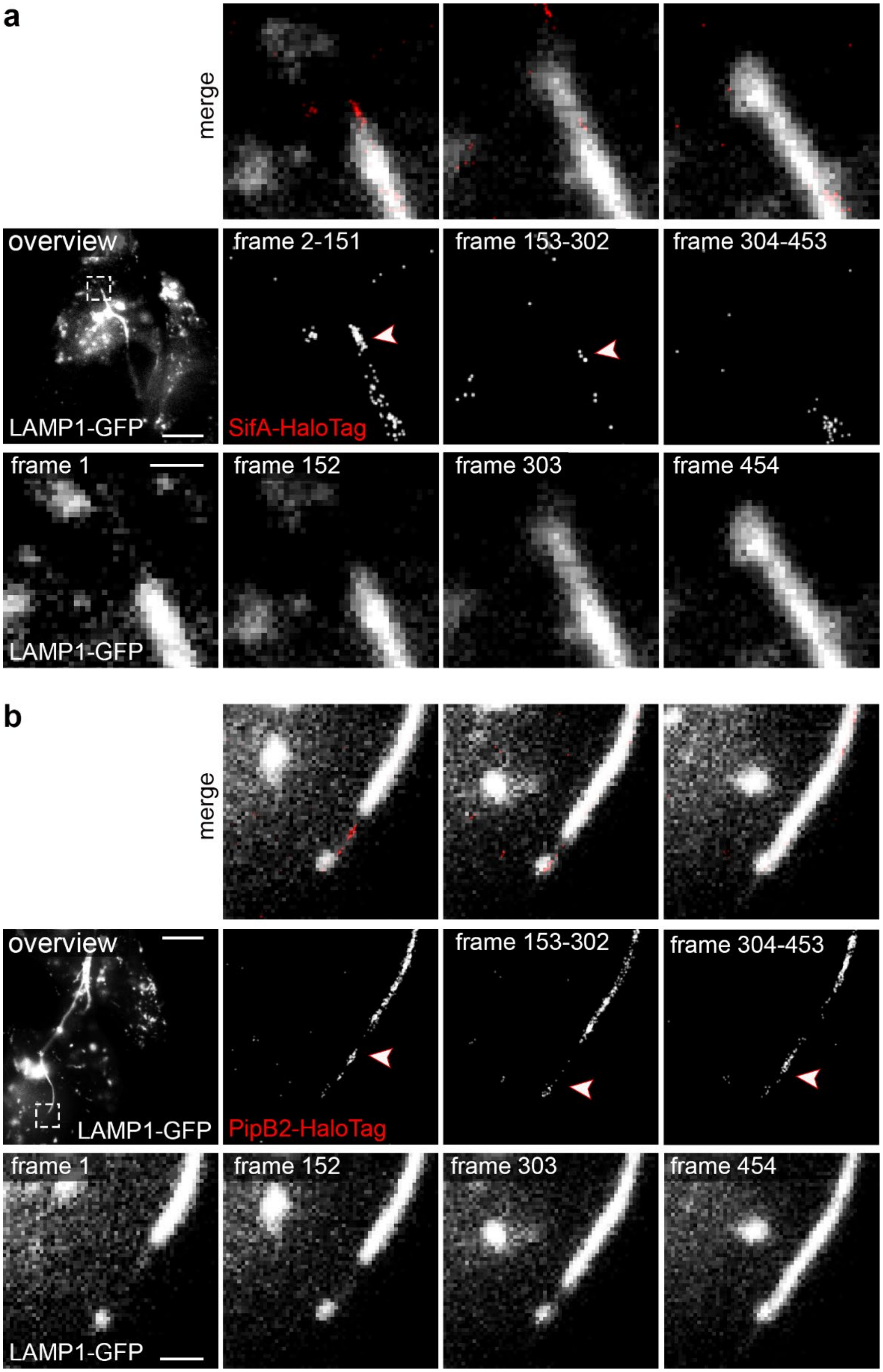

Supplementary Fig. 8. SML of effector proteins on single-membrane leading SIF. HeLa LAMP1-GFP cells were infected with STM Δ*sifA* strain expressing *sifA*::HaloTag::HA (**a**), or STM

Δ*pipB2* strain expressing *pipB2*::HaloTag::HA (**b**), and labeled with HTL-TMR as described above. The transition of leading to trailing SIF was imaged with 488 nm laser excitation for one frame (frame rate: 32 frames per second) following 561 nm laser excitation for 150 frames in 4 cycles. Shown are SRM images representative for three independent experiments, acquired using 15% laser power at the focal plane, rendered from SML within each of the 150 consecutive frames. Scale bars: 10 and 1 μm in overviews and details, respectively. The sequences of 5 frames of LAMP1-GFP are shown in Supplementary Movie 10 (SifA-HaloTag) and Supplementary Movie 11 (PipB2-HaloTag).

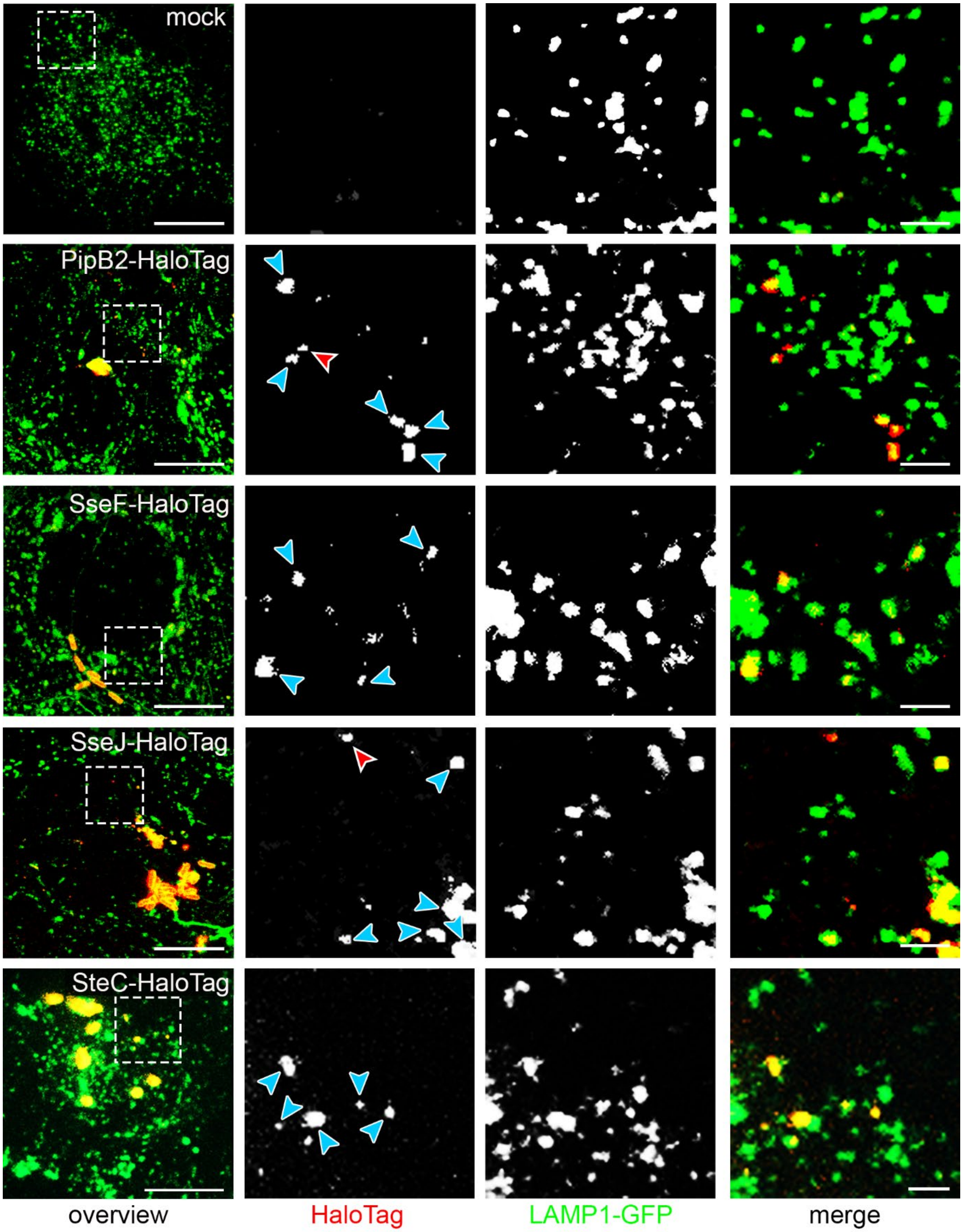

Supplementary Fig. 9. Effector protein-positive vesicles in infected HeLa LAMP1-GFP cells. HeLa LAMP1-GFP cells were infected with STM mutant strains expressing *pipB2*::HaloTag::HA, *sseF*::HaloTag::HA, *sseJ*::HaloTag::HA, or *steC*::HaloTag::HA as indicated. LCI was performed

directly after cells were stained at 3.5 h p.i. with 1 µM HTL-TMR for 30 min. Shown are representative CLSM images. Blue arrowheads indicate vesicles positive for LAMP1-GFP and effector-HaloTag labeled with TMR. Red arrowheads indicate vesicles negative for LAMP1 and positive for effector-HaloTag labeled with TMR. Micrographs show cells representative for three independent experiments. Scale bars: 10 and 2 µm in overview and details, respectively.

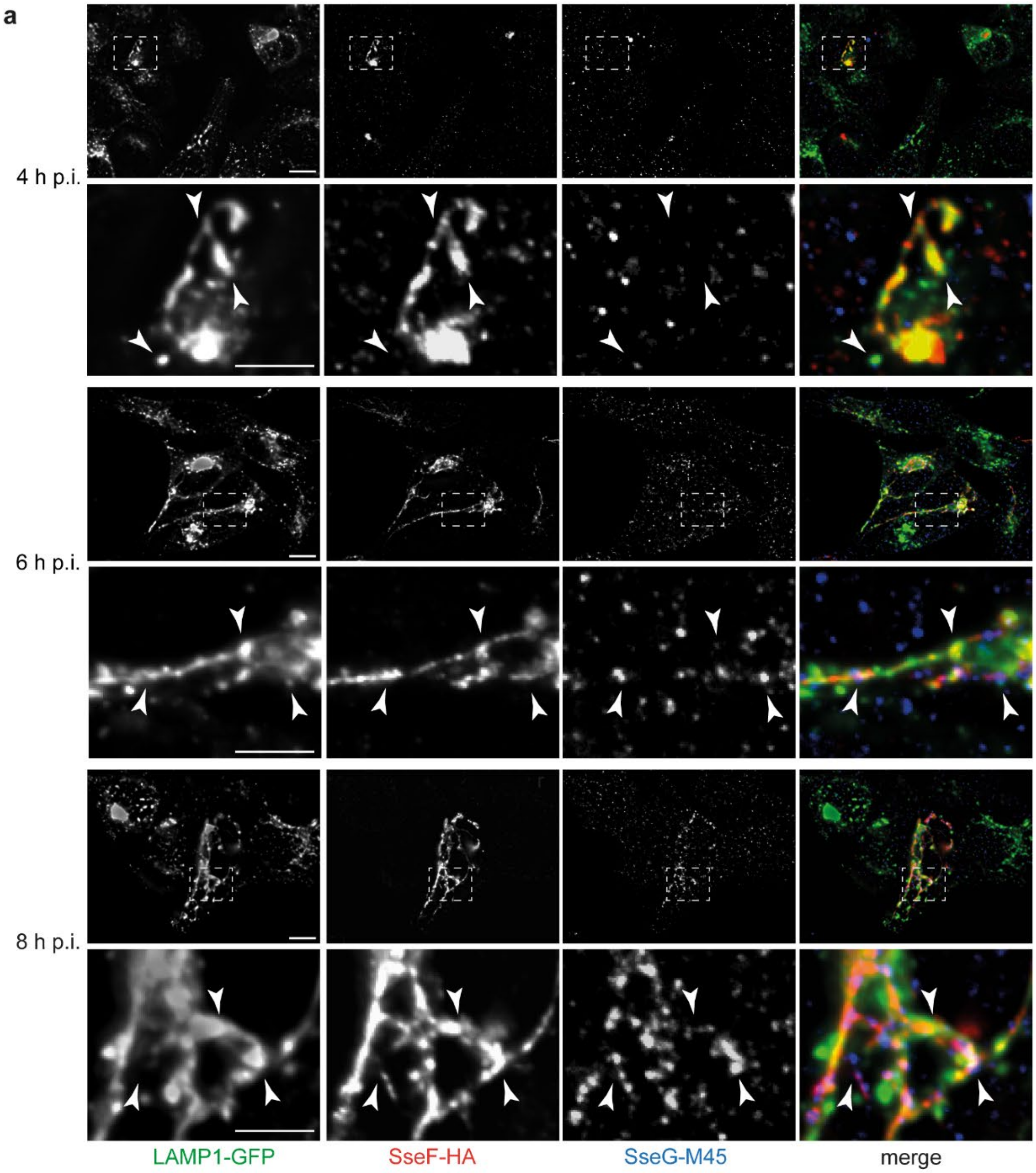

b

proteins	r 4 h	r 6 h	r 8 h
LAMP1-GFP/SseF-HA	0.286	0.594	0.447
LAMP1-GFP/SseG-M45	0.023	0.178	0.212
SseF-HA/SseG-M45	0.045	0.248	0.424

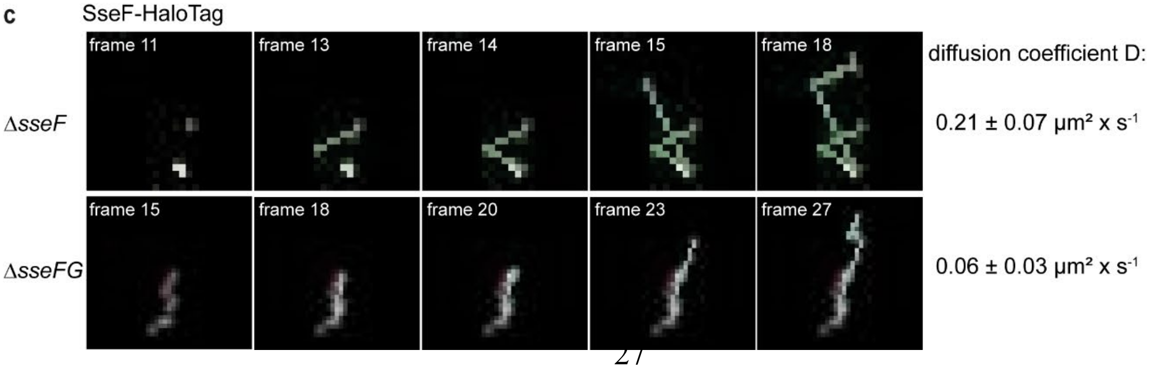

Supplementary Fig. 10. Colocalization and dynamics of effector proteins SseF and SseG. HeLa cells stably expressing LAMP1-GFP (green) were infected with STM WT expressing *sseG*::M45 *and sseF*::HA at MOI 50. **a**) At 4, 6, or 8 h p.i., cells were fixed, permeabilized and immuno-stained for HA-tagged (red) and M45-tagged (blue) effector proteins. Micrographs show cells representative for three independent experiments. Boxed ROI are shown enlarged and arrowhead indicated effector-positive vesicles. Scale bars, 10 µm (overview), 5 µm (detail). **b**) Quantification of colocalization with JACoP by using Pearson´s coefficient (r). The r values are given colocalization of SseF-HA with LAMP1- GFP, SseG-M45 with LAMP1-GFP, SseF-HA with SseG-M45. **c**) Diffusion of SseF-HaloTag is dependent on SseG. HeLa cells were infected with STM strains deficient in *sseF* (Δ*sseF*) or *sscB sseF sseG* (Δ*sseFG*) harboring a plasmid for expression of *sseF*::HaloTag. SMT was performed at 6-8 h after infection, representative tracks are shown, and diffusion coefficients were determined as described for **Fig 4.**

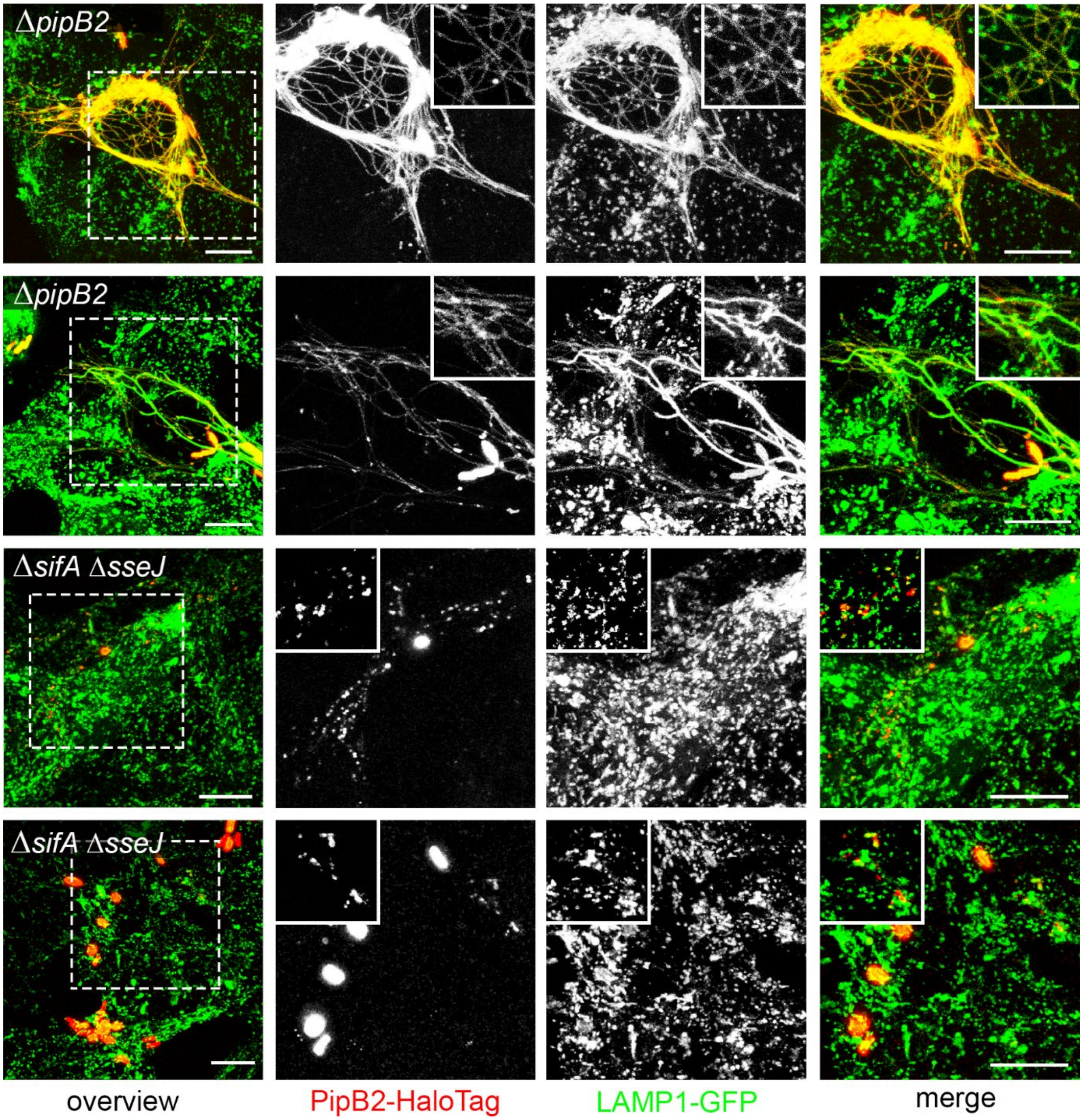

Supplementary Fig. 11. Distribution of PipB2-HaloTag in host cells infected with strains deficient in *pipB2* or Δ*sifA* Δ*sseJ*. HeLa LAMP1-GFP cells were infected with STM Δ*pipB2*, or Δ*sifA* Δ*sseJ* strains expressing *pipB2*::HaloTag::HA for 16 h. LCI was performed directly after cells were stained with HTL-TMR at a concentration of 1 µM for 30 min. Micrographs show cells representative for three independent experiments. Scale bars: 10 and 5 µm in overview and details, respectively.

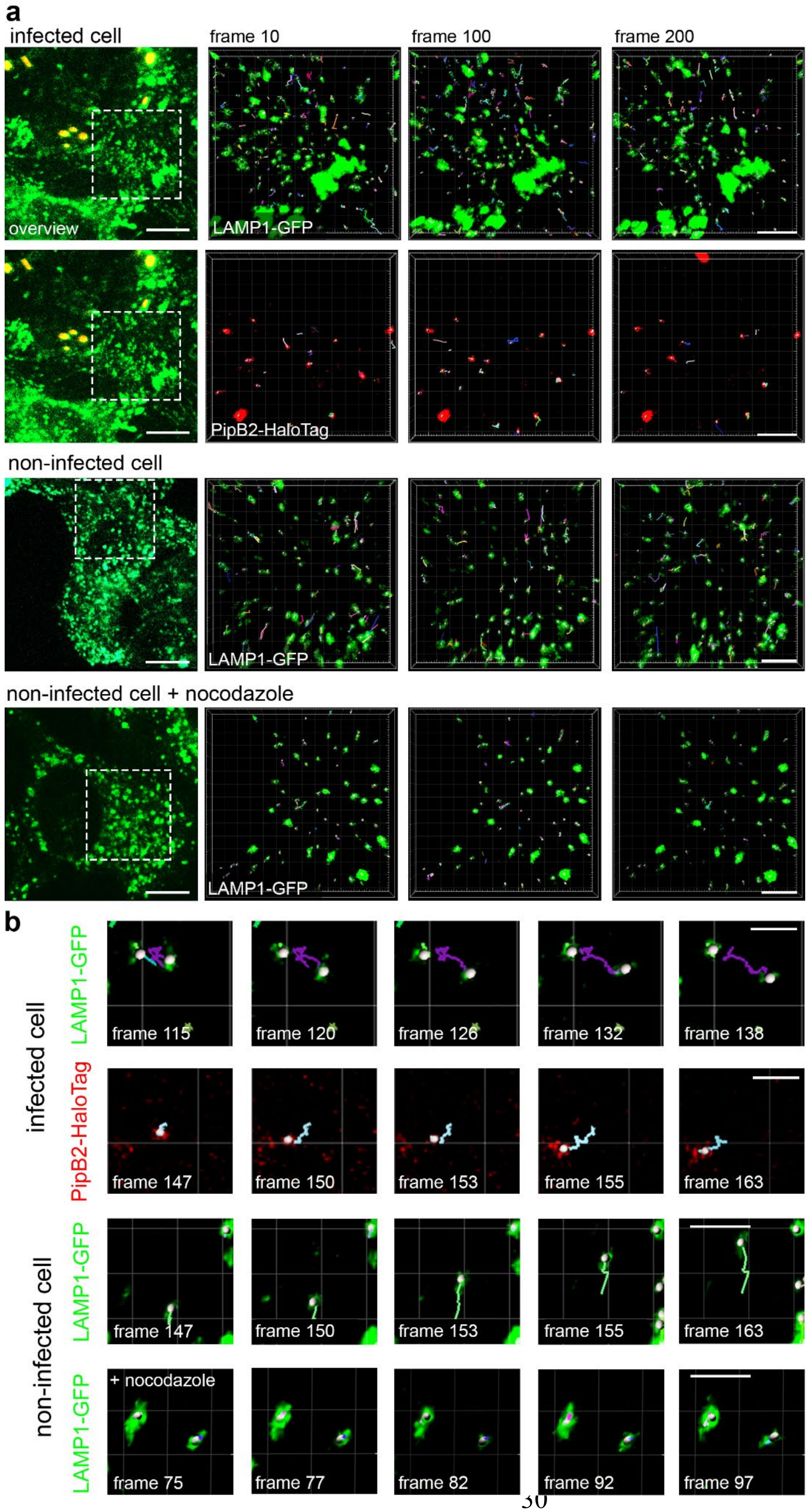
a
infected cell
frame 10
frame 100
frame 200
overview
LAMP1-GFP
PipB2-HaloTag
non-infected cell
LAMP1-GFP
non-infected cell + nocodazole
LAMP1-GFP
b
infected cell
LAMP1-GFP
frame 115
frame 120
frame 126
frame 132
frame 138
PipB2-HaloTag
frame 147
frame 150
frame 153
frame 155
frame 163
non-infected cell
LAMP1-GFP
frame 147
frame 150
frame 153
frame 155
frame 163
LAMP1-GFP
+ nocodazole
frame 75
frame 77
frame 82
frame 92
frame 97

Supplementary Fig. 12. Tracking of vesicles labeled with LAMP1-GFP and PipB2-HaloTag. HeLa LAMP1-GFP cells were either not treated, infected with STM Δ*pipB2* strain expressing *pipB2*::HaloTag::HA, or treated with nocodazole to inhibit vesicle movement. **a)** Cells were imaged for 200 frames (0.39 frames/sec) using the Zeiss SD microscope and dual camera imaging in streaming mode. At least 5 cells were imaged resulting in the analysis of at least 858 trajectories/frame. Vesicle tracking analysis was done with the Imaris spot detecting tool. See corresponding Supplementary Movies 13 and 14. **b)** Trajectories of single vesicles. Scale bars: 10 and 5 μm.
