# Supplementary material for: Single molecule analyses reveal dynamics of Salmonella translocated effector proteins in host cell endomembranes
Source: Nat Commun. 2023 Mar 4;14:1240. doi: 10.1038/s41467-023-36758-9 (PMC9985595; doi:10.1038/s41467-023-36758-9)
Supplement: Supplementary file 3 — Description of Additional Supplementary Files [file 41467_2023_36758_MOESM3_ESM.pdf]

Description of Additional Supplementary Files

File name: Supplementary Movie 1. Vesicular fusion of nanogold-labelled endosomes with the SCV/SIF continuum. HeLa cells stably expressing LAMP1-GFP (green) were infected with STM WT strain constitutively expressing GFP (green). STM cells appears as high fluorescent rods, LAMP1-GFP-positive membranes are weakly fluorescent. Gold nanoparticles were prepared by conjugating 10 nm colloidal gold with BSA-rhodamine (red). Pulse/chase with Gold nanoparticles was performed 1 h p.i. for 1 h. Live cell imaging (LCI) was performed 6 h p.i. 3D projections of image stacks were generated in Imaris. Arrows indicate fusion events nanogold-labeled endosomes to a dynamic SIF/SCV continuum.

Download link: https://myshare.uni-osnabrueck.de/f/6303fcedf52a4d42b381/

File name: Supplementary Movie 2. Single molecule tracking (SMT) of SifA-HaloTag on double-membrane SIF. HeLa cells stably expressing LAMP1-GFP were infected with STM Δ*sifA* strain expressing *sifA*::HaloTag. LCI was performed directly after labeling with 20 nM HTL-TMR. Shown are TMR signal, localization and tracking within 200 consecutive frames (corresponding to Fig. 3A).

Download link: https://myshare.uni-osnabrueck.de/f/25e95b41fd0743a1ba69/

File name: Supplementary Movie 3. SMT of LAMP1-HaloTag on double-membrane SIF. HeLa LAMP1-GFP cells were transfected with LAMP1-HaloTag one day prior infection. Cells were infected with STM WT strain and LCI was done directly after labeling with 20 nM HTL-TMR. Shown are TMR signal, localization and tracking within 200 consecutive frames (corresponding to Supplementary Fig. 2A).

Download link: https://myshare.uni-osnabrueck.de/f/2df9e8f60bb649fa9c82/

File name: Supplementary Movie 4. SMT of SseF-HaloTag on double-membrane SIF. HeLa LAMP1-GFP cells were infected with STM Δ*sseF* strain expressing *sseF*::HaloTag. LCI was done directly after labeling with 20 nM HTL-TMR. Shown are TMR signals, localization, and tracking within 200 consecutive frames (corresponding to Supplementary Fig. 2B).

Download link: https://myshare.uni-osnabrueck.de/f/3d369acf70ed4dc3bcc5/

File name: Supplementary Movie 5. SMT of PipB2-HaloTag on double-membrane SIF. HeLa LAMP1-GFP cells were infected with STM Δ*pipB2* strain expressing *pipB2*::HaloTag. LCI was done directly after labeling with 20 nM HTL-TMR. Shown are TMR signal, localization, and tracking within 200 consecutive frames (corresponding to Supplementary Fig. 2C).

Download link: https://myshare.uni-osnabrueck.de/f/f21d0afef4b543dca651/

File name: Supplementary Movie 6. SMT of PipB2-HaloTag on double-membrane SIF in fixed host cells. HeLa LAMP1-GFP cells were infected with STM Δ*pipB2* strain expressing *pipB2*::HaloTag. Cells were labeled with 20 nM HTL-TMR. Imaging was done directly after cells were fixed with 3% PFA. Shown are TMR signal, localization and tracking, within 200 consecutive frames (corresponding to Supplementary Fig. 2D).

Download link: https://myshare.uni-osnabrueck.de/f/570bade85d2c4868903c/

File name: Supplementary Movie 7. SMT of LAMP1-HaloTag on single-membrane SIF. HeLa LAMP1-GFP cells were transfected with LAMP1-HaloTag one day prior infection. Cells were infected with STM Δ*sseF* strain. LCI was done directly after labeling with 20 nM HTL-TMR. Shown are TMR signal, localization, and tracking within 200 consecutive frames (corresponding to Fig. 4A).

Download link: https://myshare.uni-osnabrueck.de/f/1c42b10e1f414ab0a3b3/

File name: Supplementary Movie 8. SMT of SifA-HaloTag on single-membrane SIF. HeLa LAMP1-GFP cells were infected with STM Δ*sseF* Δ*sifA* strain expressing *sifA*::HaloTag. LCI was performed directly after labeling with 20 nM HTL-TMR. Shown are TMR signal, localization, and tracking within 200 consecutive frames (corresponding to Fig. 4B).

Download link: https://myshare.uni-osnabrueck.de/f/919d7993597e40948b9b/

Movie 9. Localization of SseF-HaloTag during transition of leading to trailing SIF. HeLa LAMP1-GFP cells were infected with STM Δ*sseF* strain expressing *sseF*::HaloTag and labeled with HTL-TMR. The transition of leading to trailing SIF was imaged with 488 nm laser excitation for one frame, following 561 nm laser excitation for 150 frames in 4 cycles. Shown are 5 frames of 488 nm excitation (corresponding to Fig. 4E).

Download link: https://myshare.uni-osnabrueck.de/f/2cb53caa8c81451c89a4/

File name: Supplementary Movie 10. Localization of SifA-HaloTag during transition of leading to trailing SIF. HeLa LAMP1-GFP cells were infected with STM Δ*sifA* strain expressing *sifA*::HaloTag and labeled with HTL-TMR. The transition of leading to trailing SIF was imaged with 488 nm laser excitation for one frame, following 561 nm laser excitation for 150 frames in 4 cycles. Shown are 5 frames of 488 nm excitation (corresponding to Supplementary Fig. 3A).

Download link: https://myshare.uni-osnabrueck.de/f/4851e1aaa09b437aa0a4/

File name: Supplementary Movie 11. Localization of PipB2-HaloTag during transition of leading to trailing SIF. HeLa LAMP1-GFP cells were infected with STM Δ*pipB2* strain expressing *pipB2*::HaloTag and labeled with HTL-TMR. The transition of leading to trailing SIF was imaged with 488 nm laser excitation for one frame,

following 561 nm laser excitation for 150 frames in 4 cycles. Shown are 5 frames of 488 nm excitation (corresponding to Supplementary Fig. 3A).

Download link: https://myshare.uni-osnabrueck.de/f/777f93142ae7459ca5fc/

File name: Supplementary Movie 12. Co-tracking of LAMP1-GFP- and PipB2-HaloTag-positive vesicles in infected host cells. HeLa LAMP1-GFP cells were infected with STM Δ*pipB2* strain expressing *pipB2*::HaloTag. LAMP1-positive- and PipB2-HaloTag-positive vesicles were imaged and vesicle tracking analysis was done with Imaris spot detection tool. Shown are 200 frames of co-tracking of vesicles positive for LAMP1 and PipB2 co-tracking (corresponding to Fig. 6A).

Download link: https://myshare.uni-osnabrueck.de/f/095efdca37b14916ad6a/

File name: Supplementary Movie 13. Tracking of LAMP1-GFP- and PipB2-HaloTag-positive vesicles in STM-infected host cells. HeLa LAMP1-GFP cells were infected with STM Δ*pipB2* strain expressing *pipB2*::HaloTag. LAMP1-positive and PipB2-positive vesicles were tracked with the Imaris spot detection tool in individual cells over 200 frames (corresponding to Fig. 6A).

Download link: https://myshare.uni-osnabrueck.de/f/2bc6b6801d8240449e47/

File name: Supplementary Movie 14. Tracking of LAMP1-GFP-positive vesicles in non-infected and nocodazole-treated cells. HeLa LAMP1-GFP cells either non-treated or treated with nocodazole. LAMP1-positive vesicles were tracked with the Imaris spot detection tool in individual cells over 200 frames (corresponding to Fig. 6A).

Download link: https://myshare.uni-osnabrueck.de/f/a633c24d92f24442a4a2/

File name: Supplementary Movie 15. PipB2-HaloTag distribution in STM-infected cells. HeLa LAMP1-GFP cells were infected with STM Δ*pipB2* strain expressing *pipB2*::HaloTag. Time-lapse imaging was performed directly after cells were stained with HTL-TMR (1 µM). Cells were imaged every 30 min starting 5 h p.i. until 12 h p.i. (corresponding to Fig. 7A).

Download link: https://myshare.uni-osnabrueck.de/f/5e80ad49973a43e8a9d5/

File name: Supplementary Movie 16. Effect of nocodazole on PipB2-HaloTag distribution in STM-infected cells HeLa LAMP1-GFP cells were infected with STM Δ*pipB2* strain expressing *pipB2*::HaloTag. Cells were treated with nocodazole (2 h p.i.). Time-lapse imaging was performed directly after cells were stained with HTL-TMR (1 µM) and inhibitor was removed. Cells were imaged every 30 min starting 5 h p.i. until 12 h p.i. (corresponding to Fig. 7AB).

Download link: https://myshare.uni-osnabrueck.de/f/55645ebb2c4a4b89a1aa/
