# Supplementary material for: Single molecule analyses reveal dynamics of Salmonella translocated effector proteins in host cell endomembranes
Source: Nat Commun. 2023 Mar 4;14:1240. doi: 10.1038/s41467-023-36758-9 (PMC9985595; doi:10.1038/s41467-023-36758-9)
Supplement: Supplementary file 20 — Reporting Summary [file 41467_2023_36758_MOESM20_ESM.pdf]

nature portfolio

Corresponding author(s): Michael Hensel

Last updated by author(s): Feb 2, 2023

Reporting Summary

Nature Portfolio wishes to improve the reproducibility of the work that we publish. This form provides structure for consistency and transparency in reporting. For further information on Nature Portfolio policies, see our Editorial Policies and the Editorial Policy Checklist.

Statistics

For all statistical analyses, confirm that the following items are present in the figure legend, table legend, main text, or Methods section.

n/a	Confirmed	
☐	☒	The exact sample size (*n*) for each experimental group/condition, given as a discrete number and unit of measurement
☐	☒	A statement on whether measurements were taken from distinct samples or whether the same sample was measured repeatedly
☐	☒	The statistical test(s) used AND whether they are one- or two-sided<br>*Only common tests should be described solely by name; describe more complex techniques in the Methods section.*
☐	☒	A description of all covariates tested
☐	☒	A description of any assumptions or corrections, such as tests of normality and adjustment for multiple comparisons
☐	☒	A full description of the statistical parameters including central tendency (e.g. means) or other basic estimates (e.g. regression coefficient) AND variation (e.g. standard deviation) or associated estimates of uncertainty (e.g. confidence intervals)
☐	☒	For null hypothesis testing, the test statistic (e.g. *F*, *t*, *r*) with confidence intervals, effect sizes, degrees of freedom and *P* value noted<br>*Give P values as exact values whenever suitable.*
☒	☐	For Bayesian analysis, information on the choice of priors and Markov chain Monte Carlo settings
☒	☐	For hierarchical and complex designs, identification of the appropriate level for tests and full reporting of outcomes
☒	☐	Estimates of effect sizes (e.g. Cohen's *d*, Pearson's *r*), indicating how they were calculated

Our web collection on statistics for biologists contains articles on many of the points above.

Software and code

Policy information about availability of computer code

Data collection	ZEN Core microscopy software (Zeiss) versions 3.2<br>Leica LAS-AG microscopy software (Leica), version AF 2.6.7266
Data analysis	FIJI (ImageJ package), version 2.9<br>MatLab 2013a<br>SlimFast, code for the application is available under: https://github.com/CPaoloR/SLIMfast<br>SigmaPlot 13<br>Adobe Creative Suite 6<br>OMERO.server: 5.6.4<br>OMERO.insight: 5.7.1<br>OMERO.web: 5.14.1<br>BioFormats version: 6.10.0

For manuscripts utilizing custom algorithms or software that are central to the research but not yet described in published literature, software must be made available to editors and reviewers. We strongly encourage code deposition in a community repository (e.g. GitHub). See the Nature Portfolio guidelines for submitting code & software for further information.

Data

Policy information about availability of data

All manuscripts must include a data availability statement. This statement should provide the following information, where applicable:

- Accession codes, unique identifiers, or web links for publicly available datasets
- A description of any restrictions on data availability
- For clinical datasets or third party data, please ensure that the statement adheres to our policy

the data will be available via a repository upon acceptance

Human research participants

Policy information about studies involving human research participants and Sex and Gender in Research.

Reporting on sex and gender	not applicable
Population characteristics	not applicable
Recruitment	not applicable
Ethics oversight	not applicable

Note that full information on the approval of the study protocol must also be provided in the manuscript.

Field-specific reporting

Please select the one below that is the best fit for your research. If you are not sure, read the appropriate sections before making your selection.

☒ Life sciences ☐ Behavioural & social sciences ☐ Ecological, evolutionary & environmental sciences

For a reference copy of the document with all sections, see nature.com/documents/nr-reporting-summary-flat.pdf

Life sciences study design

All studies must disclose on these points even when the disclosure is negative.

Sample size	n.a.
Data exclusions	No data were excluded from the analyses
Replication	All experimental analyses were performed in at least three independent biological replicates. At least three successful independent biogical replicates were included in data analyses
Randomization	Randomization was not applicable, since Salmonella-infected cells with or without further manipulation had to be identified by microscopy by the investigator. After recording micrographs or time lapse series. data analyses were performed the the number of data sets described in the publication.
Blinding	Blinding was not applicable, since Salmonella-infected cells with or without further manipulation had to be identified by microscopy by the investigator. After recording micrographs or time lapse series. data analyses were performed the the number of data sets described in the publication.

Reporting for specific materials, systems and methods

We require information from authors about some types of materials, experimental systems and methods used in many studies. Here, indicate whether each material, system or method listed is relevant to your study. If you are not sure if a list item applies to your research, read the appropriate section before selecting a response.

Materials & experimental systems

n/a	Involved in the study	
☐	☒	Antibodies
☐	☒	Eukaryotic cell lines
☒	☐	Palaeontology and archaeology
☒	☐	Animals and other organisms
☒	☐	Clinical data
☒	☐	Dual use research of concern

Methods

n/a	Involved in the study	
☒	☐	ChIP-seq
☒	☐	Flow cytometry
☒	☐	MRI-based neuroimaging

Antibodies

Antibodies used

Mouse anti M45 IgG (custom-made by production from hybridoma line)
Rat anti HA c3 F10 IgG (Roche #1 1867431001 by Merck)
Mouse anti HA c16B12 IgG, (Roche # 93019429)
Rabbit anti Salmonella O, group B factors 1, 4, 5, 12 (BD Difco # 228151)
Alexa Fluor 568 goat anti mouse (Invitrogen # A-11004 by ThermoFisher)
Goat anti rat IgG HRP (Jackson ImmunoResearch # 112-035-003)
Goat anti rabbit IgG Cy5 (Jackson ImmunoResearch # 111-175-144)
Alexa Fluor 568 goat anti rat IgG (H+L) (Life Technologies # A-11077 by ThermoFisher)
Protein A gold 10 nm (custom-made by Cell Microscopy Core, University Medical Center, Utrecht, NL)

Validation

Primary antibodies were validated by binding to HA- or M45-tagged proteins in Western Blots and epifluorescecne microscopy. Non-tagged variants of the proteins served as controls. Antiserum against Salmonella was validated by slide agglutination tests with group B Salmonella

Eukaryotic cell lines

Policy information about cell lines and Sex and Gender in Research

Cell line source(s)

RAW264.7 (American Type Culture Collection, ATCC no. TIB-71)
HeLa (ATCC no. CCL-2)
lentiviral transfected RAW264.7 and HeLa constitutively expressing hLAMP1-eGFP

Authentication

lentiviral transfected RAW264.7 and HeLa were authentified by epifluorescence microscopy for constitutive expression of hLAMP1-eGFP

Mycoplasma contamination

cell llines were tested on regular base for Mycoplasma contamination and confirmed negative

Commonly misidentified lines
(See ICLAC register)

no commonly misidentified cell lines were used in the study.
